# Prevalence and clinical features of adverse food reactions in Portuguese adults

**DOI:** 10.1186/s13223-016-0139-8

**Published:** 2016-08-05

**Authors:** Carlos Lozoya-Ibáñez, Sara Morgado-Nunes, Alexandra Rodrigues, Cláudia Lobo, Luis Taborda-Barata

**Affiliations:** 1 Castelo Branco Local Health Unit, Allergy Department, Castelo Branco, Portugal; 2 Faculty of Health Sciences, CICS-Health Sciences Research Centre, University of Beira Interior, Avenida Infante D. Henrique, 6200-506 Covilhã, Portugal; 3 Polytechnic Institute of Castelo Branco, Escola Superior de Gestão, Castelo Branco, Portugal; 4 Castelo Branco Local Health Unit, Outpatient Department, Castelo Branco, Portugal; 5 Castelo Branco Local Health Unit, Clinical Pathology Department, Castelo Branco, Portugal; 6 Department of Allergy and Clinical Immunology, Cova da Beira Hospital Centre, Covilhã, Portugal

**Keywords:** Adverse food reaction, Food allergy, Adults, Prevalence, Cutaneous tests, Open food challenge

## Abstract

**Background:**

Only one previous study, via telephone call, on the prevalence of self-reported food allergies has been performed in Portugal, in a small sample of adults. The objective of this study was to determine the prevalence of self-reported and probable food allergy, analyze the clinical features and involved foods in Portuguese adults.

**Methods:**

Population-based, cross-sectional study performed in various healthcare centres from central Portugal. All 1436 randomly selected individuals (median age: 45 years, 50.6 % female) replied to a validated food allergy questionnaire by phone. Those who reported an adverse food reaction were invited to come to the hospital, where clinical history was taken, skin prick (SPT) and prick-prick skin (SPPT) tests were performed and food allergen-specific IgE levels (sIgE) were determined. An open oral challenge was performed in selected cases. Cases of positive clinical history of immediate (up to 2 h after ingestion) reaction in association with positive food sIgE levels and/or skin prick tests were classified as IgE-associated probable food allergy. Cases of positive clinical history of delayed (more than 2 h after ingestion) and negative food sIgE levels independently of positive SPT or SPPT results were classified as non-IgE associated probable food allergy.

**Results:**

The prevalence of probable food allergy in our sample was 1 %, with shellfish and fish as the most frequently implicated foods. IgE-mediated probable food allergy occurred in 0.71 % of cases, with shellfish, peanut and nuts mainly involved. Cutaneous symptoms were most frequently reported. Prevalence values and food types were discrepant between self-reported and probable food allergies.

**Conclusions:**

The prevalence of probable food allergies in Portuguese adults is low, is mostly related to shellfish, peanut and nuts and most frequently involves cutaneous symptoms.

**Electronic supplementary material:**

The online version of this article (doi:10.1186/s13223-016-0139-8) contains supplementary material, which is available to authorized users.

## Background

Food allergy is an important health problem in western-style countries, as the high number of publications (around 21,000 in the past 10 years) on this issue seems to indicate [1, 2]. Although the prevalence of food allergies is not as high as that of other allergic diseases, its repercussions on dietary habits and social integration of food allergic patients is quite relevant [3, 4]. In this regard, a study from the US has shown that about 20 % of the American population changes their diet due to an adverse food reaction, namely food allergy [5]. Furthermore, the economic impact of food allergies, namely in terms of work absentism, is quite high and has been estimated to average around 510 million US$ per year in the US [6].

However, not all adverse reactions to foods are regarded as having an immunologically mediated “food allergy” [1, 7–9]. It is, in fact, necessary to go through a complicated diagnostic process, involving a thorough and detailed clinical history as well as specific tests, among which oral challenges are included [7, 9, 10]. If the diagnostic process is not completed to a great extent, or is somehow incorrect, it may lead to unnecessary or inappropriate dietary eviction measures.

Partly for this reason, the prevalence values of food allergies in the general adult population are not well known. Various meta-analyses [11, 12] have estimated the prevalence of food allergies to any food between 3.5 and 35 % when only self-reported values are analysed and between 2 and 4 % when studies include diagnostic tests. However, these values seem to vary according to the country of reference and the methodology used. Thus, in the US, a recent study based upon a detailed review of various studies that analysed self-reported symptoms as well as confirmed food allergies [13], estimated that food allergies affect “more than 1–2 % but less than 10 %” of the population. In Europe, two population-based studies based upon questionnaires applied via telephone call and followed by clinical assessment, skin prick tests and oral challenge tests, clearly showed discrepant results between the prevalence values of self-reported and confirmed food allergy in adults in Germany (34.9 % self-reported and 3.7 % confirmed food allergy) [14], and in Denmark (19.6 % self-reported and 1.7 % confirmed food allergy) [15], values which are relatively similar to those obtained in the US. As far as we know, no population-based studies on the prevalence of food allergies have been carried out in Portugal, with the exception of one study on self reported food allergy, via telephone call, in a small sample of adults from the city of Oporto [16]. Thus, the objective of our study was to determine the prevalence of both self-reported and probable food allergy, as well as to analyze the clinical features and involved foods in a general population of Portuguese adults.

## Methods

### Population

For this study, we took into account the fact that 76,946 adults of both sexes, aged between 18 and 80 years, are registered in the files of general practitioners from the six Healthcare Centres belonging to the Local Health Unit of Castelo Branco which accepted to participate in the study (Castelo Branco, Vila Velha de Ródão, Sertã, Proença-a-Nova, Oleiros and Idanha-a-Nova). This is representative of a sample of the general population, since all Portuguese citizens are covered by the National Health Service/Care, and are thus registered at a Healthcare Centre, where they are assigned to a specific general practitioner.

Based on an estimated prevalence of 4 % [12, 14, 16], and considering a 95 % confidence interval and a margin of error of 2 % we calculated that we would need a representative sample of 369 adults. Considering an expected reply rate of 40 %, the sample size was set at 923 adults. We therefore decided to contact at least 1000 adults (about 1.3 % of total population) proportionally distributed in accordance with the number of individuals registered at each Healthcare Centre, located in both rural and urban areas, and randomly selected to be contacted by telephone.

### Study design

**Fig. 1:**
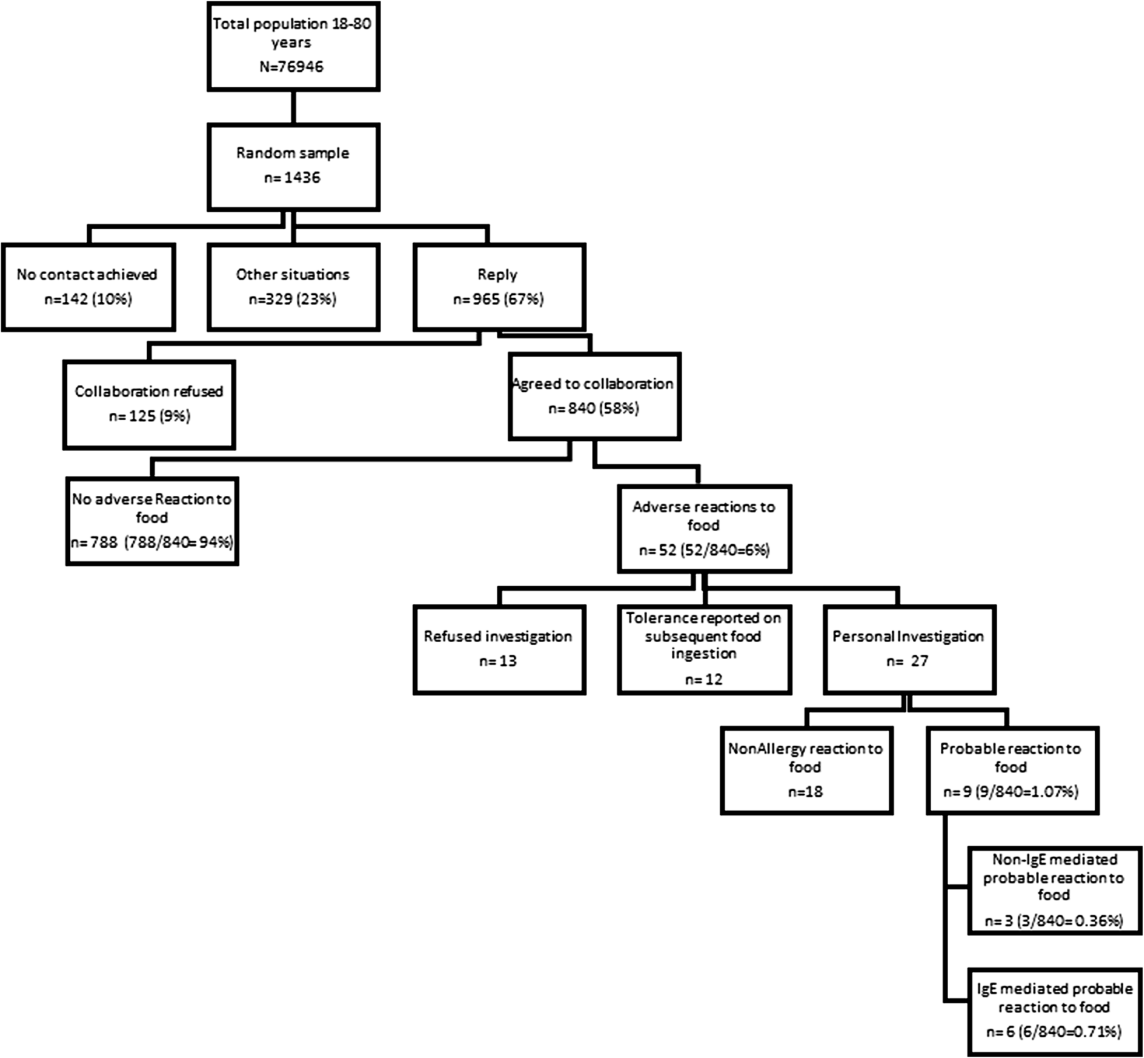
Flow chart of the study design and investigations performed

Population-based, cross-sectional study, performed in a 2 year-long period (2013–2014). It was approved by the Ethics Committees of the Amato Lusitano Hospital and the former Administrative Sub-Region of Health of Castelo Branco. All patients gave written informed consent. All 1436 randomly selected individuals (mean age: 47 years, median age: 45 years, 50.6 % female) registered at participating Healthcare Centres were contacted by telephone and a validated food allergy questionnaire was applied [17]. More specifically, a code number was given to each adult individual registered at each Healthcare centre. Randomization was carried out using a specific randomizer programme (http://www.socialpsychology.org/randomizer.htm), following the principles of simple random sampling. All individuals were contacted by phone in up to three attempts and would not be included in the study if these attempts failed. Those who reported a previous adverse food reaction were invited to participate in the rest of the study (Fig. 1). These volunteers were subsequently contacted by a specialist doctor, and those who again confirmed the persistence of an adverse food reaction were invited for an appointment at the Outpatient Allergy Clinic of the Amato Lusitano Hospital, where a standardized food allergy-related clinical history was taken [18], skin prick tests (SPT) and, where applicable, prick-prick skin tests (SPPT) were performed and blood was collected for determination of food allergen-specific IgE levels. In those cases in which the clinical history was not clear and SPT results as well as specific IgE levels were negative, an open oral challenge was performed. If these patients did not exclude the suspected food from the diet, an eviction diet was followed for a minimum of 7 days prior to the food challenge. Patients with a positive clinical history of immediate (up to 2 h after ingestion) reaction in association with positive food sIgE levels and/or skin prick tests were classified as IgE-associated probable food allergy. Patients with a positive clinical history of delayed (more than 2 h after ingestion) and negative food sIgE levels independently of positive SPT or SPPT results were classified as non-IgE associated probable food allergy.

### Questionnaire

A 17-item, previously validated questionnaire on adverse food reactions [17] (Additional file 1) was applied by phone to all volunteers. This questionnaire included demographic data, questions on the occurrence of previous episodes of adverse reactions to foods, types of foods causing such episodes, types of reactions, post-ingestion latency time for appearance of symptoms, date of latest reaction, need for medical assistance, personal or family history of atopic diseases.

### Determination of levels of allergen-specific IgE

In all individuals who came to the outpatient clinic, 5 ml of peripheral blood was taken for the determination of the levels of total serum IgE, aeroallergen-specific screening IgE (Phadiatop inhalant allergens^®^), as a marker of atopy and suspected food-specific IgE. A fluorometric (ImunoCAP^®^ 250 Phadia Diagnosis)-based technique was used (Phadia and Thermo Scientific, Uppsala, Sweden). Allergen-specific levels above 0.35 KU_A_/L were regarded as positive.

### Skin Prick Tests

In vivo studies included SPT (LETI Laboratories, Spain; Bial-Aristegui, São Mamede do Coronado, Portugal; Stallergènes, Antony, France) for aeroallergens (house dust mites, cockroach, fungi, latex, cat and dog dander, weeds, tree and grass pollens) and suspected foods and/or SPPT with the suspected foods. Tests were carried out in duplicate on the volar aspect of the forearms. A drop of each commercial extract was placed upon the skin and each drop was pricked through using a metal lancet (Stallergènes, Antony, France). The mean weal diameter was recorded after 15 min. Wheals with a mean diameter at least 3 mm greater than that of the negative control were regarded as positive. SPPT tests used the same methodology.

### Oral challenge

Oral challenges were performed in all cases with unclear clinical history independently of positive or negative SPT [7, 9, 10]. In those cases in which individuals did not avoid the foodstuffs, in spite of having symptoms, an eviction diet for at least 7 days before the oral challenge was carried out and monitored. Oral challenge was performed in an open manner, at the hospital, under direct clinical observation for 4 h post-challenge and further 24 h-long monitoring, depending upon presence or absence of reported symptoms. No double-blind, placebo-controlled food challenges were carried out.

### Statistical analysis

Data was analysed using the Software Package for Social Sciences (SPSS) version 20.0^®^ (SPSS Inc., Chicago, IL, USA). Analysis of normality of distribution of variables was performed using the One Sample Kolmogorov–Smirnov test. Descriptive analysis was used for the characterization of the sample. Chi Square test or Fischer’s Exact Test were used in the case of nominal variables. Comparative analysis of quantitative variables was carried out using Student’s t test or Mann–Whitney U test depending on distribution of variables. Odds ratio values were calculated for analysis of possible risk factors for adverse for reactions. A *p* value of less than 0.05 was regarded as significant with all statistical tests.

## Results

### Determination of prevalence and features of self-reported food allergy

Of the 1436 randomly selected individuals, we successfully contacted 965 by telephone (67 % reply rate), and the questionnaire was fully completed in 840 cases (58 % of the total sample). These individuals had a mean age of 48 years (median age: 46 years), and 51.3 % were female. Most individuals belonged to Graffar scale classes III and IV, without significant differences in comparison with individuals who declined to participate in the study (data not shown). Furthermore, participants and those who declined to participate were similar in terms of mean age and gender.

Of these, 52 reported previous adverse reaction upon ingestion of at least one foodstuff, giving an estimated prevalence of 6 % (95 % CI 4.4–7.6 %) (Fig. 1). The self reported reactions had mostly occurred in the 6 months to 5 years previous to the phone contact (n = 35; 42 % of the cases).

Most commonly reported foods were seafood (34.6 %), various fresh fruits (21.1 %) and fish (19.2 %).

**Fig. 2:**
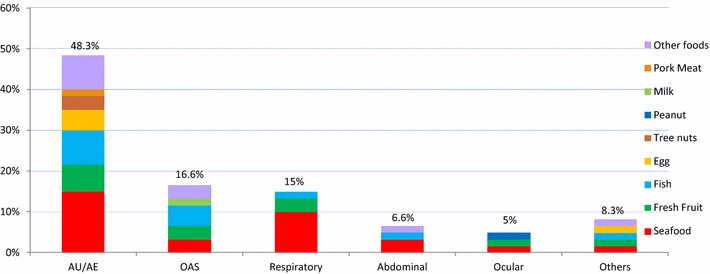
Self-reported symptoms associated with each food type (percentage of cases)

**Fig. 3:**
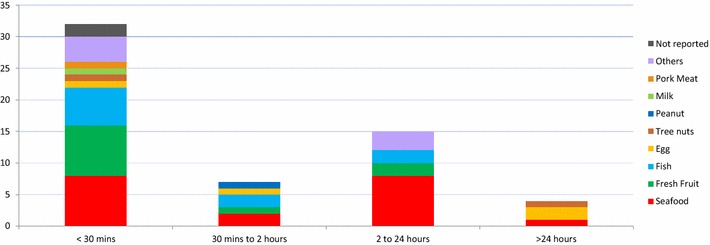
Time for development of symptoms upon food ingestion (number of cases)

**Fig. 4:**
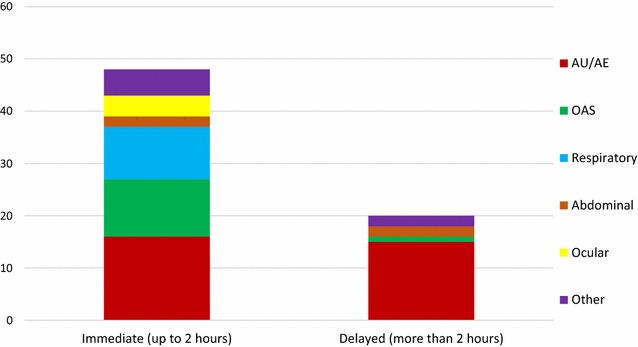
Types of reactions observed, in relation to the timeframe of their development upon ingestion of food (immediate versus delayed), (number of cases)

Most frequently reported symptoms were cutaneous—urticarial and/or angioedema (48.3 % of the cases), followed by oral allergy syndrome (OAS) (16.6 %), respiratory (15 %) and gastro-intestinal/abdominal—dyspepsia, abdominal pain, diarrhea and/or vomiting (6.6 %) symptoms (Fig. 2). In most cases (55 %), symptoms developed within 30 min upon ingestion and only 26 % of the cases had a delayed onset (between 2 and 24 h) (Fig. 3). The different types of reactions observed, in relation to the timeframe of their development upon ingestion of food (immediate versus delayed), are shown in Fig. 4.

**Fig. 5:**
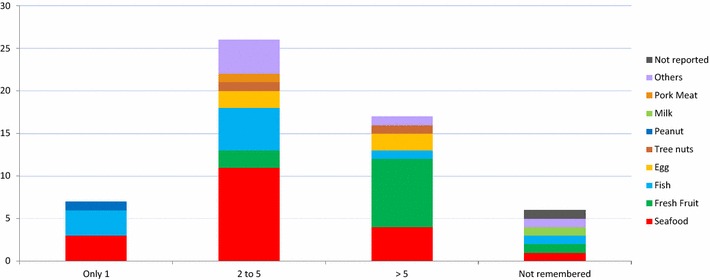
Number of episodes with the same food (number of cases)

Most individuals reported between two and five episodes with the same food (46.6 %, with seafood being the most frequent one). More than five episodes were reported in 31 % of the cases, with fresh fruits being the food most frequently involved (Fig. 5).

Medical treatment had been given in 29/52 (56 %) of the cases. Most individuals (27/52; 51 %) had never been diagnosed an adverse food reaction, and only 16 % (**8**/**51**) had been diagnosed a food allergy by an Allergist.

Having a personal (OR 3.72; 95 % CI 2.04–6.77) or a family history (OR 1.70; 95 % CI 0.90–3.21) of atopy were factors significantly associated with an increased risk of having an adverse food reaction.

### Determination of prevalence and features of probable food allergy

Of the 52 cases who reported AFR, 13 (25 %) declined to continue in the study, and 39 were invited to the hospital. We obtained information from all of these individuals (75 % of all AFR cases). Of these, 18 (35 %) reported that they had tolerated the suspected food after the initial phone call, and 21 individuals (40 % of the total of AFR) completed the full study (clinical history, SPT/SPPT and determination of total and allergen-specific IgE levels).

Upon analysis of the clinical history, laboratory data and SPT/SPPT results, an oral challenge test was carried out when there were doubts regarding the presence of a food allergy. Four open oral challenges were performed in two volunteers. One of the challenges was clearly positive (angioedema of the face, tongue and lips starting 15 min upon the beginning of the challenge) but the remaining challenges were negative. The patient with the positive oral challenge (patient #9) refused a new challenge with the other implicated food (Table 1). This patient was regarding as having non-IgE associated food reaction since she had negative food-specific IgE levels, low total serum IgE levels, negative personal and family history of atopy and her reported reactions upon ingestion of the suspect food were delayed.

**Table 1:** Characteristics of diagnosed food allergic patients

Patient ID	#1	#2	#3	#4	#5	#6	#7	#8	#9
Age	37	57	34	36	50	47	55	37	58
Gender	M	M	F	F	M	M	F	F	F
IgE levels (KU_A_/L)	114	540	255	128	125	82	36	26	30
Food-specific IgE	Pos	Pos	Pos	Neg	Pos	Pos	Neg	Neg	Neg
Personal history of atopy	Yes	No	Yes	Yes	No	No	Yes	No	Yes
Family history of atopy	Yes	Yes	Yes	Yes	No	No	Yes	No	No
Phadiatop	Pos	Pos	Pos	Pos	Neg	Pos	Neg	Neg	Neg
SPT aeroallergens	Pos	Pos	Pos	Pos	Pos	Pos	Neg	Neg	Neg
Sensitization to >1 food	No	No	No	Yes	No	Yes	No	No	No
Foods	Shellfish	Shellfish	Shellfish	Fruits, seafood	Shellfish	Peanut, tree nuts	Fish	Shellfish	Fish
Manifestations	Asthma	Anaphylaxis	AU/AE	OAS	AU/AE	AU/AE	AU/AE	AU/AE	AU/AE
Time until symptom development	<30 min	<30 min	<30 min	<30 min	30 min–2 h	30 min–2 h	2–24 h	2–24 h	2–24 h
SPT with commercial food extracts	Pos	Pos	Pos	Pos	Pos	Pos	Neg	Pos	Neg
Prick by Prick food skin test	Pos	Pos	Pos	Pos^a^	Pos	Pos	Pos	Pos	Neg
Open food challenge	Not performed	Not performed	Not performed	Not performed	Not performed	Not performed	Not performed	Not performed	Pos
Probable allergy mechanism	IgE-mediated	IgE-mediated	IgE-mediated	IgE-mediated	IgE-mediated	IgE-mediated	Non IgE-mediated	Non IgE-mediated	Non IgE-mediated

*M* male; *F* female; *Pos* positive; *Neg* negative; *AU* acute urticaria; *AE* angioedema; *OAS* oral allergy syndrome

^a^Positive only to seafood

An immunologically-mediated adverse food reaction was diagnosed in 9 patients [9/840; 1 % (95 % CI 0.39–1.31 %)] of the total of number of individuals (mean age: 45 years, median age: 47 years, 55.6 % female). IgE-mediated sensitization was demonstrated in 6 of them, giving a value of probable food allergy of 0.71 % (95 % CI 0.14–1.28 %). The details of the patients who were regarded as having immunologically mediated food allergy are shown in Table 1. Most frequently implicated foods were shellfish (50 %) and fish (20 %). IgE-mediated sensitization was only detected in four of the six cases, in association with the ingestion of shellfish and in 1 case, with the ingestion of nuts and peanut. Two individuals had reactions with more than one food, but IgE-mediated sensitization was only shown in one volunteer who was allergic to peanut and nuts.

Of the six cases in which an IgE-associated mechanism was detected, Phadiatop was positive in five, whereas this test was negative in all cases of food allergy in which IgE-mediated sensitization was not shown. In addition, the values of total serum IgE were significantly higher in the group of patients with demonstrated IgE-mediated sensitisation, as compared with the group with non-IgE-mediated reactions (207.33 KUA versus 30.66 KUA, respectively; p < 0.001; Mann–Whitney U test).

SPT performed with food commercial extracts were positive with seven out of nine foods reported in the IgE-mediated group, in comparison with only two out of five foods reported in the non-IgE-mediated group (general sensitivity of test of 64 %, specificity of 82 %, PPV: 64 %, NPV: 82 %).

SPPT carried out with fresh foods were positive in eight out of nine cases in the volunteers from the IgE-mediated group and in three out of five volunteers of the non IgE-mediated group (general sensitivity of the test of 89 %, specificity: 79 %, PPV: 66 %, NPV: 94 %).

In terms of symptoms reported in cases diagnosed as probable food allergy (both IgE- and non-IgE-mediated), the most prevalent one was cutaneous (50 % of cases), followed by respiratory (22 %) and OAS (22 %). Delayed symptoms, occurring between 2 and 24 h upon ingestion, were only reported in three out of nine cases, all of which belonging to the non IgE-mediated group. In the remaining six cases, reactions were immediate, and all occurred in individuals from the IgE-mediated group. Of all the 27 individuals observed at the Hospital, about 57 % reported that they had needed treatment for their food-induced symptoms.

## Discussion

The objective of our work was to determine, for the first time in Portugal, the prevalence of probable food allergy, the type of implicated foods, types of symptoms and other associated factors in a general adult population. We have shown that the prevalence of probable food allergies in this population is low, is mostly related to shellfish, peanut and nuts and most frequently involves cutaneous symptoms.

The utilization of a questionnaire by phone call showed that the prevalence of self-reported food allergy in our study (6 %) was within values reported in other population-based studies namely in Europe, the US and Canada (between 3 and 19 %) [11, 12, 14, 16, 19–23]. On the other hand, the prevalence of probable food allergy in our study (1 %), based upon a positive clinical history, positive skin prick tests and/or food-specific IgE levels and, in some cases, also on a single-blinded food challenge was lower than that observed in North American adults (between 1 and 10 %) [13], but fell within the range of results of various European studies (between 0.8 and 1.1 %) [12, 15, 20].

The discrepancy in prevalence data between self-reported symptoms and symptoms confirmed by medical evidence (in vitro and in vivo tests and/or oral challenges) has been reported by various groups [11, 12, 14, 15, 20, 21]. Curiously, in a study carried out in Canada, no significant differences were observed between self-reported symptoms with a set of five foods [22] and the subsequent confirmation of food allergies [23], but the methodology was different. Thus, most studies have shown that self-reported symptoms tend to overestimate the prevalence of food allergies, and suggest that this may be partly explained by a bias in self perception of symptoms and wrongly ascribing them to the ingestion of foods. Cultural or health literacy factors, or low accessibility to medical services may also be involved, since, in our study, only 16 % of those individuals who were contacted had previously consulted a specialist doctor because of their symptoms. Nevertheless, differences in prevalence values across studies are hardly comparable, given the heterogeneity of methodologies followed and adult populations included.

The types of foods most frequently implicated in our study, both in self-reported and in probable allergy cases, are included in the so-called “big eight allergens”—milk, egg, peanut, tree nuts, wheat, soy, fish and shellfish [24] and are similar to those found in studies using similar methodologies in Europe [12, 14, 15, 19, 20], namely in Southern Europe [16, 25, 26], the US [7, 9, 21, 27] and Canada [22]. However, the individual prevalence of each food type was different in our study, which may be due to cultural differences in food habits, although we cannot exclude the possibility that the lower size of our sample in comparison with some of the other studies may have influenced the results. In addition, in contrast with our study, the OAS is not always regarded as a symptom of food allergy since it is frequently associated with pollinosis and is regarded as a “secondary allergy” [15, 20, 28].

We also detected discrepancies in implicated foods between the self-reported results (shellfish, fresh fruits and fish) and those obtained upon allergological testing (shellfish, fish, peanut and nuts), as has been previously reported [14, 15, 19, 20]. Furthermore, various meta-analyses [11, 12, 28] have also identified such a discrepancy, and ascribe it to differences in concepts between adverse food reactions perceived by the individual and the immunologically-based “allergy” diagnosed by an allergist. These observations stress the need for an adequate diagnostic approach in order to avoid unnecessary diets [3, 4]. Furthermore, a confirmed diagnosis of food allergy may also increase awareness for prevention of accidental contacts with allergenic foods [29–32].

Cutaneous (urticarial and/or angioedema) manifestations were the most prevalent clinical manifestations both in self-reported and in confirmed, probable food allergy-related cases, as has been previously described [7, 9, 12, 14–16, 19–22, 25, 27], although that was not the case in a questionnaire-based study in the UK [33]. However, in this study, only a limited repertoire of foods was analysed, which may explain this discrepancy.

Analysis of self-reported symptoms found associations between ingestion of certain foods and the development of symptoms. Shellfish, fruits and fish were associated with cutaneous manifestations and OAS, and shellfish and fruit were most frequently associated with respiratory and abdominal symptoms. Fish and shellfish were most frequently triggers of single and more severe episodes (mostly involving respiratory symptoms).

In addition, we also found two different, time-related predominant response patterns, previously identified by Osterballe [20]: an immediate type, developing in up to 30 min post-ingestion, mainly associated with shellfish, fresh fruits and fish, and a delayed type, occurring between 2 and 24 h upon ingestion, with shellfish as the principal implicated food type. The reason underlying this difference is not clear, although it may have been due to discrepant IgE-binding capacity of B cell epitopes on different food allergens [34].

In patients with confirmed probable food allergy, we observed an inverse association between symptom development latency time and their severity. We did not find any case with a latency time greater than 24 h, independently of the pathophysiological mechanism involved, as previously reported [20].

We also performed multivariate analysis of the association between various risk factors and the development of food allergies. Although the relatively small size of our simple may have biased the analysis, personal and family history of allergies were significantly associated with food allergies, which is in agreement with previous studies [1, 7, 12, 35].

Although we performed open food challenges in some of the patients, it was not possible to perform double blind, placebo-controlled food challenges, which are regarded as the “gold standard” for the final diagnosis of food allergies. This was a weakness of our methodology. In spite of this limitation, our approach included not only a standardized clinical history, but also the application of a validated questionnaire, SPT/SPPT, determination of food-specific IgE levels and open oral challenges in certain cases, which makes it a thorough study. In fact, many of the various population studies on food allergies performed in other countries only applied a questionnaire [8, 19, 21–23], and a few others only added skin tests and/or determination of food-specific IgE levels in cases with suspected food allergy [26, 36].

One of the strengths of our study is that we were able to obtain information from most (75 %) of those individuals who had reported a food allergy, indicating that our study was associated with a relatively low drop out rate, which might, otherwise, be a limiting factor. In fact, it has been described that participation rate seems to be inversely related to the thoroughness of a study, averaging between 31–67 % [8, 14, 20] when only questionnaires are involved but dropping to around 40 % [14] when volunteers are requested to undergo a more thorough assessment. In view of this, having had a drop out rate of 25 % in our study allowed us to meet the necessary calculated requirements for representativeness and statistical proportionality.

## Conclusions

The prevalence of probable food allergy in Portuguese adults was low, around 1 %, with shellfish and fish as the most frequently implicated foods. IgE-mediated probable food allergy occurred in 0.71 % of the cases, with shellfish, peanut and nuts mainly involved. Cutaneous symptoms were most frequently reported. There was a discrepancy between self-reported and probable food allergies, both in terms of prevalence values but also in terms of implicated foods.

Our study significantly contributes towards the study of food allergies in Portugal, and it may also be a useful tool for comparison with other studies carried out in other countries.

## Additional file

10.1186/s13223-016-0139-8 Validated questionnaire used for assessing adverse food reactions in Portuguese adults.

## Abbreviations

AFR: adverse food reaction
AU/AE: acute urticaria/angioedema
CI: confidence interval
IgE: immunoglobulin E
NPV: negative predictive value
OAS: oral allergy syndrome
OR: odds ratio
PPV: positive predictive value
SPPT: skin prick by prick test
SPT: skin prick test
