# Supplementary material for: Prevalence and clinical features of adverse food reactions in Portuguese adults
Source: Allergy Asthma Clin Immunol. 2016 Aug 5;12:36. doi: 10.1186/s13223-016-0139-8 (PMC4975906; doi:10.1186/s13223-016-0139-8)
Supplement: Supplementary file 1 — 10.1186/s13223-016-0139-8 Validated questionnaire used for assessing adverse food reactions in Portuguese adults. [file 13223_2016_139_MOESM1_ESM.pdf]

Additional File

Validated Questionnaire used for assessing adverse food reactions in Portuguese adults

Table 1: Questionnaire

Question Number	Item
1	Identity Code of volunteer
2	Gender
3	Age in years
4	Do you want to answer this questionnaire?
5	Do you have any adverse food reaction?
6	What kind of food causes your reaction?
7	What kind of reaction did you have?
8	How long after food ingestion did the reactions appear?
9	Did you need medical treatment?
10	If answer was “yes” for item 9, Where did you receive medical treatment?
11	Have you had any previous episodes with the same food?
12	How long ago did the previous reaction take place?
13	Have you been previously diagnosed a food allergy?
14	Have you ever been to a specialty appointment by an Allergist doctor?
15	Do you have any other allergic disease? (personal history of atopy)
16	Does anybody in your family have an allergic disease?
17	Would you want to be followed up at a specialty clinic?
